# Insight into Two ABC Transporter Families Involved in Lantibiotic Resistance

**DOI:** 10.3389/fmolb.2017.00091

**Published:** 2018-01-22

**Authors:** Rebecca Clemens, Julia Zaschke-Kriesche, Sakshi Khosa, Sander H. J. Smits

**Affiliations:** Institute of Biochemistry, Heinrich-Heine-University Duesseldorf, Duesseldorf, Germany

**Keywords:** lanthionine ring, lantibiotic, nisin, resistance, antimicrobial peptide, *L. lactis*

## Abstract

Antimicrobial peptides, which contain (methyl)-lanthionine-rings are called lantibiotics. They are produced by several Gram-positive bacteria and are mainly active against these bacteria. Although these are highly potent antimicrobials, some human pathogenic bacteria express specific ABC transporters that confer resistance and counteract their antimicrobial activity. Two distinct ABC transporter families are known to be involved in this process. These are the Cpr- and Bce-type ABC transporter families, named after their involvement in cationic peptide resistance in *Clostridium difficile*, and bacitracin efflux in *Bacillus subtilis*, respectively. Both resistance systems differentiate to each other in terms of the proteins involved. Here, we summarize the current knowledge and describe the divergence as well as the common features present in both the systems to confer lantibiotic resistance.

## Introduction

The urging need for novel antibiotics has put small antimicrobial peptides (AMPs) into a particular focus. Especially, a large group of peptides called bacteriocins have been extensively studied for an application purpose as novel antibiotics. Bacteriocins are small, ribosomally-synthesized peptides of which some display a high potent antimicrobial activity (Tagg et al., 1976; Cotter et al., 2005b) and have been already used since decades as food preservatives or as antibiotic alternatives in biomedical applications (Cleveland et al., 2001; Cotter et al., 2012).

**Figure 1:**
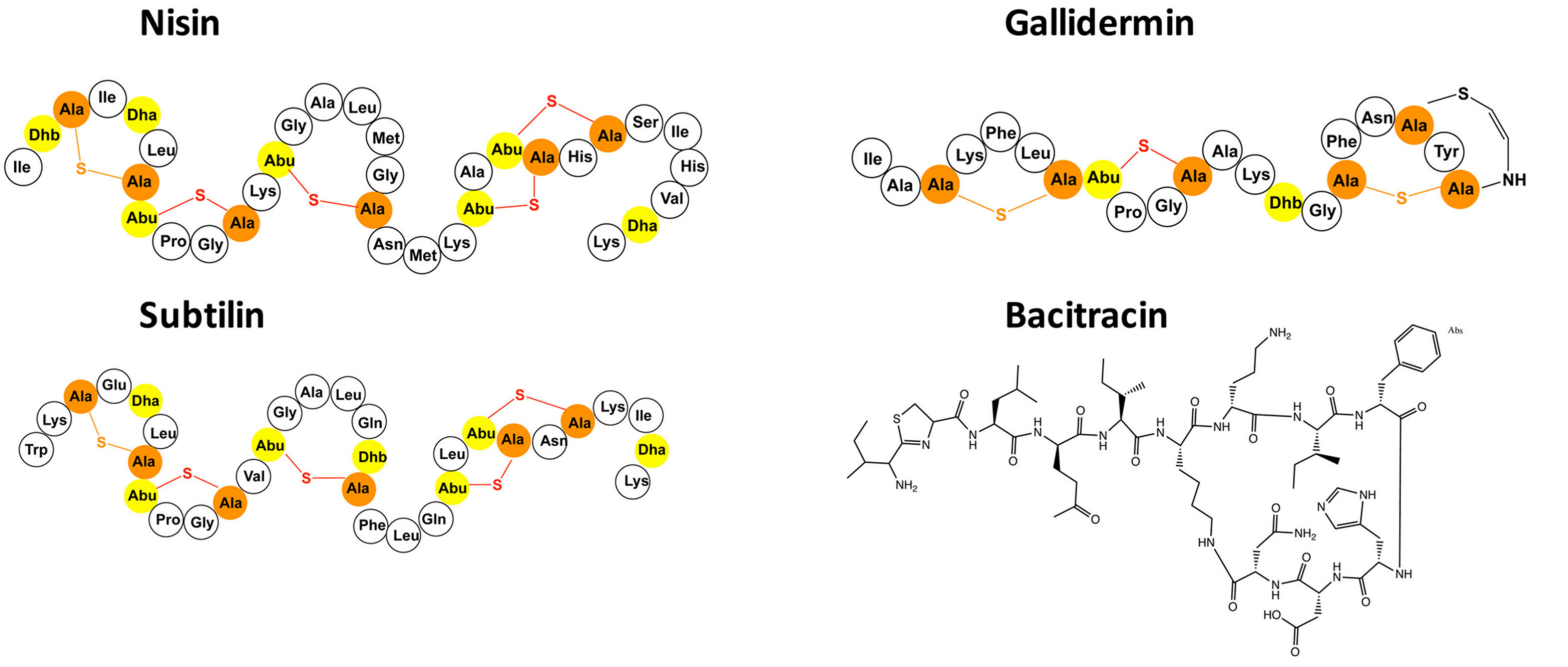
Selected presentation of the lantibiotics nisin, gallidermin and subtilin as well as the bacteriocin bacitracin. The dehydrated amino acids and the cysteines of the lantibiotics are highlighted in yellow and orange. The (methyl-)lanthionine rings are visualized in orange and red.

A large group within the bacteriocin family, are ***lan***thionine containing *an****tibiotics*** termed lantibiotics. These lantibiotics are post-translationally modified peptides that contain dehydrated amino acids (Dehydrobutyrine and/or Dehydroalanine) and other unusual amino acid modifications (Jung, 1991; Willey and van der Donk, 2007; Bierbaum and Sahl, 2009; Alvarez-Sieiro et al., 2016). The Michael addition of a neighboring cysteine side chain residue to these dehydrated amino acids results in the formation of characteristic thioether bridges called lanthionine rings. These rings are primarily crucial for their high antimicrobial activity against mainly Gram-positive bacteria. The well-known lantibiotics nisin, gallidermin, and subtilin are highlighted in Figure 1. Lantibiotics are highly potent and nanomolar concentrations are already enough to fulfill their antimicrobial activity as observed for example for nisin produced by *Lactococcus lactis* species or subtilin produced by *Bacillus subtilis* (Delves-Broughton et al., 1996; Chatterjee et al., 2005).

In comparison to their high antimicrobial activity against Gram-positive bacteria, lantibiotics display a reduced effectiveness against Gram-negative bacteria. Many lantibiotics bind to lipid II or other peptidoglycan precursor inducing inhibition of cell wall synthesis. Some lantibiotics can subsequently form pores which lead to membrane leakage and rapid cell death (Héchard and Sahl, 2002; Bierbaum and Sahl, 2009). Due to their nanomolar activity, in combination with high stability against proteolytic digestion, lantibiotics are considered potential compounds for novel medical treatment.

A well-studied member of lantibiotics is nisin, which is produced by some *L. lactis* strains. It was shown that it is effective against the treatment of bacterial mastitis, methicillin-resistant *Staphylococcus aureus* (MRSA) and enterococcal infections (Brumfitt et al., 2002). Gallidermin and epidermin, produced by *Staphylococcus gallinarum* and *Staphylococcus epidermidis*, respectively, are some other examples of lantibiotics (Cotter et al., 2005a) and are associated with the treatment of acne, eczema, folliculitis, and impetigo.

The lantibiotic producer strains with a few exceptions, usually contain a single gene cluster, on which the structural genes for the lantibiotic itself, as well as for the modification and transport across the cellular membrane are located (Chatterjee et al., 2005; Willey and van der Donk, 2007; Alkhatib et al., 2012; Singh and Sareen, 2014). In many gene clusters, these genes are upregulated via a distinct two-component system (TCS) consisting of a histidine kinase (HK) and a response regulator (RR), which are located on the same gene cluster (Qiao et al., 1996). The upregulation is auto induced by its own lantibiotic (Kuipers et al., 1995).

Due to the high convergence of these gene clusters, it has been possible to detect potential lantibiotic gene clusters within newly sequenced genomes using *in silico* techniques (van Heel et al., 2013a). Such genome mining approaches have identified novel lantibiotic gene clusters in several species, such as the genes encoding for maddinglicin from *Clostridium maddingley*, agalacticin from *Streptococcus agalactiae*, bagelicin from *Streptococcus suis* and moraviensicin from *Enterococcus moraviensis* (van Heel et al., 2013b; Tracanna et al., 2017). These novel and mostly exotic lantibiotics can be expressed, modified and secreted by *L. lactis* using the well-characterized nisin biosynthetic machinery (van Heel et al., 2013b).

In order to prevent the activity of the secreted lantibiotic against their own membrane, the lantibiotic gene cluster contains additional genes (*lanI* and *lanFEG*), which form a lantibiotic (auto-)immunity system (Alkhatib et al., 2012). The *lanI* and *lanFEG* genes are conserved to a certain extent throughout the lantibiotic expressing bacteria (Alkhatib et al., 2012). Here LanI is a membrane-associated lipoprotein, which binds to the lantibiotic and thereby lowers the concentration of the lantibiotic reaching the membrane. Additionally, LanFEG forms an ABC transporter localized in the cellular membrane which effluxes the lantibiotic prior to pore formation (Stein et al., 2003, 2005; Draper et al., 2008, 2015).

Despite the odds, resistance against lantibiotics does exist and different resistance mechanisms have been unraveled so far. Resistance mechanisms comprise of modification in peptidoglycan or the cellular membrane (e.g., changes in phospholipid or fatty acid composition) as well as cell membrane modifications, such as lipopolysaccharides which are attached to the outer layer of the outer membrane of Gram-negative bacteria (Draper et al., 2015). Furthermore, some TCSs could be linked to lantibiotic resistance by upregulating the transcription of resistance-associated genes upon the presence of lantibiotic within the habitat of the bacteria. Other mechanisms resulting in resistance are the assembly of biofilms or the expression of resistance proteins such as the nisin resistance protein (NSR) found to be upregulated in nisin non-producing strains, inactivating nisin by specific proteolytic degradation (Sun et al., 2009). The lantibiotic resistance mechanisms have been nicely reviewed in detail in Draper et al. (2015).

Recently, several gene clusters were identified in various human pathogenic bacteria, which encode a lantibiotic resistance system based on the overexpression of membrane embedded proteins, that includes the presence of an ABC transporter (Khosa et al., 2013).

The expression of proteins within these gene clusters result in a detectable lantibiotic resistance. For example, resistance against nukacin ISK-I and lacticin 481 in *Streptococcus mutans* is mediated by the expression of *lcrSR-lctFEG* genes (Kawada-Matsuo et al., 2013a), while the expression of *cprABCK-R* operon in *Clostridium difficile* results in resistance against different lantibiotics. Here, nisin, mutacin 1140, subtilin, and gallidermin were tested and resistance was observed (McBride and Sonenshein, 2011; Suárez et al., 2013). Furthermore, the proteins located on the *nsr* operon from *S. agalactiae* are together conferring resistance against nisin A, nisin H, and gallidermin (Khosa et al., 2016a,b; Reiners et al., 2017). All these resistance operons are characterized by the presence of a TCS consisting of a HK and a RR; as well as a membrane-embedded ATP-binding cassette (ABC) transporter (Gebhard, 2012; Khosa et al., 2013; Suárez et al., 2013). In some cases, an extra gene encoding a membrane-associated lipoprotein or a specific serine protease is present.

Upon examining these operons in detail, it was observed that the ABC transporters are responsible for lantibiotic resistance and can be divided into two groups: the CprABC-type and the BceAB-type ABC transporter family, both conferring resistance against lantibiotics and/or antimicrobial peptides in general.

Within this review, we will highlight these two lantibiotic resistance ABC transporter families and their corresponding gen clusters.

## Gene cluster organization of ABC transporters involved in lantibiotic resistance

**Figure 2:**
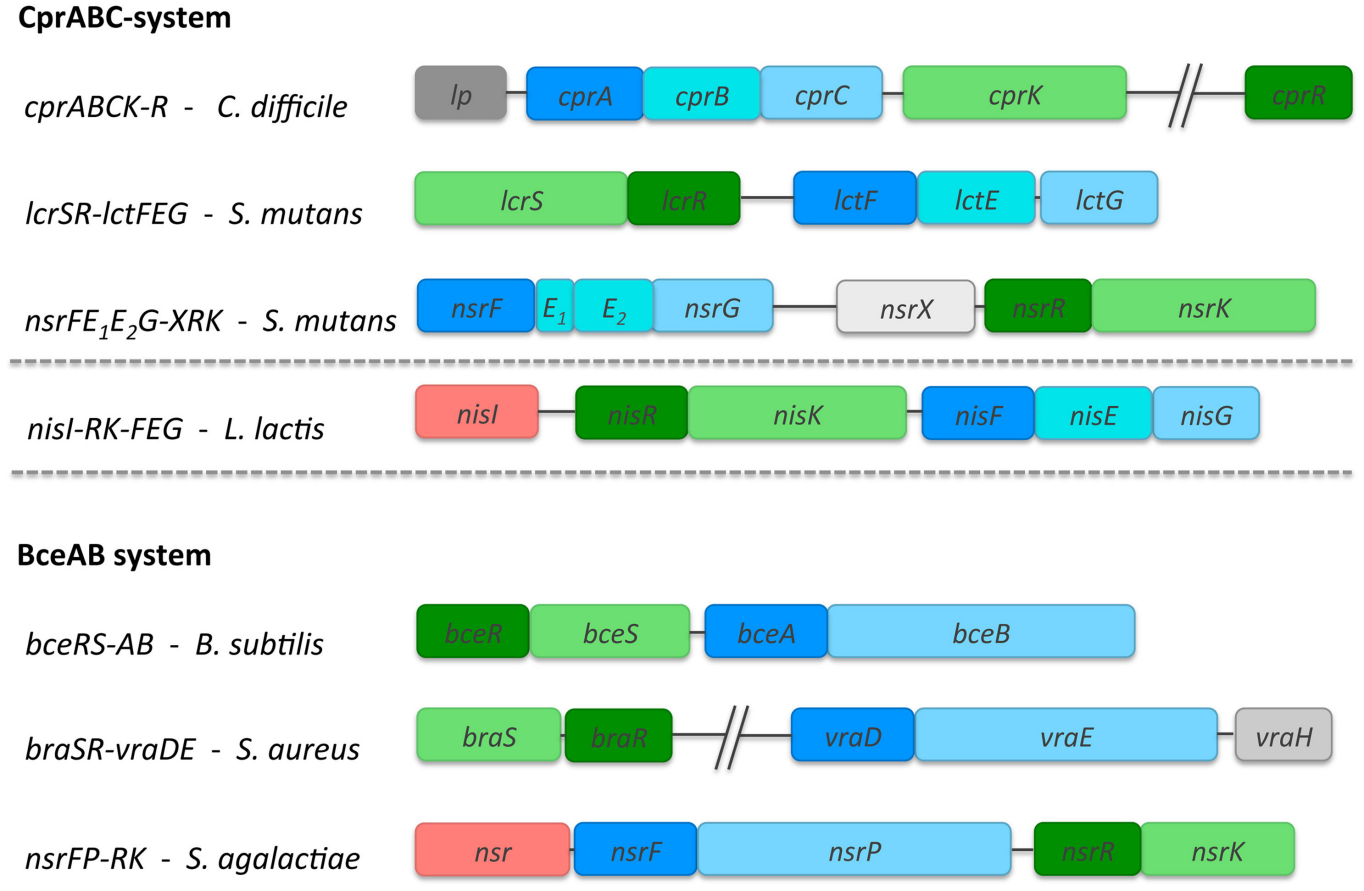
Overview of the lantibiotic resistance operons belonging to the Cpr- and Bce- type systems. Three operon structures, each are highlighted as representatives for the Cpr and Bce group. For Cpr-type these are *cprABCK-R* from *C. difficile, nsrFE*_1_*E*_2_*G-XRK* and *lcrSR-lctFEG* from *S. mutans*. Additionally, the *nisRK-FEG* system from *L. lactis* involved in nisin immunity is also highlighted. For Bce-type, the three representatives comprise of *bceRS-AB* from *B. subtilis, braSR-vraDE* from *S. aureus* and the *nsrFP-RK* system from *S. agalactiae*. The size of the genes corresponds directly with the gene length as deposited in the NCBI database. The TCSs with RR (dark green) and HK (light green); and the ABC transporters are shown in different shades of blue. In the *bceAB* system, the NBD is dark blue while the TMD is shown in light blue. The additional TMD present in the *cprABC* systems is shown in cyan. The proteins, which are part of the operon but the function has not been determined so far are shown in gray. In case of the *nis* and *nsr* operons, an additional membrane-associated protein is present which is colored in red.

The common feature of both operon types is the presence of a TCS, which upregulates the other genes by an external stimulus via binding of the lantibiotic. Furthermore, they both consist of an ABC transporter, which is thought to expel the lantibiotic once it has reached the bacterial membrane (Figure 2). In general, ABC transporter comprises of a transmembrane domain (TMD) and a nucleotide-binding domain (NBD). The NBD dimerizes upon binding of ATP, which is subsequently hydrolysed and the energy released is used to induce a conformational change within the TMD allowing substrate export or import. Furthermore, in some of these operons there is also the presence of either a lipoprotein or a membrane-associated specific protease.

## CprABC-type resistance operons

**Figure 3:**
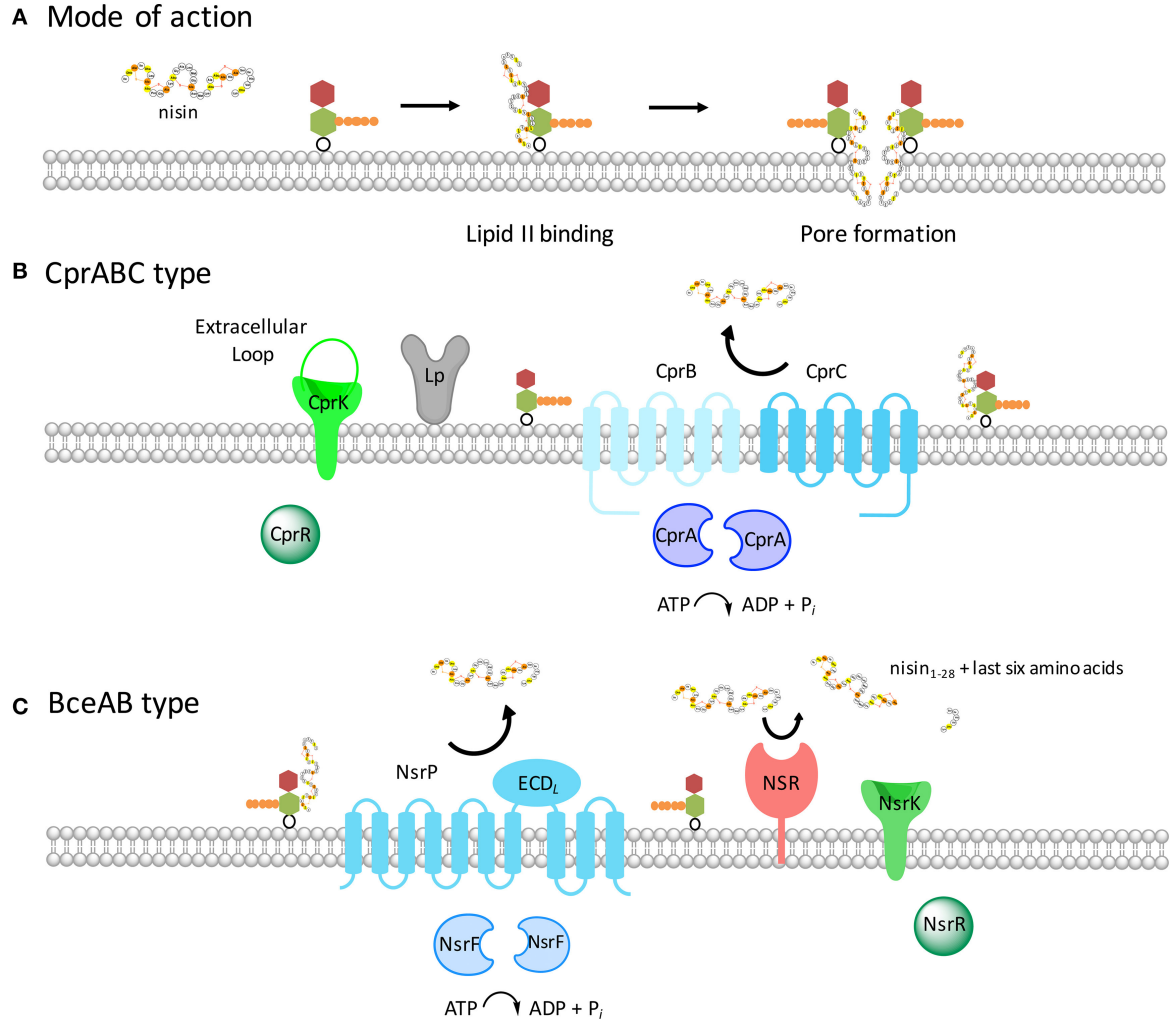
Schematic representation of the two resistance systems of *C. difficile* and *S. agalactiae*. **(A)** The lantibiotic nisin A (see Figure 1) binds to the cell wall precursor lipid II, depicted with the N-acetyl glucosamine colored in red, the N-acetyl muramic acid shown in green and the pentapeptide presented in orange. The binding via the first two lanthionine rings of nisin to lipid II results growth inhibition and subsequently in pore formation. This results in rapid cell death already at nanomolar nisin concentrations. **(B)** The proteins encoded by the *cpr* operon are shown with the ABC transporter CprABC (depicted in blue) and the two-component system CprRK (depicted in green). The extracellular loop of the histidine kinase has also been shown. The lipoprotein adjacent to CprABC is depicted in gray. **(C)** The Nsr system of *S. agalactiae* is highlighted. The two-component system NsrRK and an efflux ABC transporter NsrFP are depicted in green and blue, respectively. The ECD_*L*_ present in the ABC transporter in between transmembrane helices seven and eight is indicated in cyan. Furthermore, an extra membrane-associated serine protease *Sa*NSR present in this system is shown in red. *Sa*NSR is a serine protease, which cleaves the last six amino acids of nisin off.

Lantibiotic resistance operons belonging to the *Cpr* group contain three different genes encoding for an ABC transporter (one for the NBD and two different TMDs; highlighted in blue, Figure 2) and genes encoding a HK and RR, which build up the TCS. These transporters belong to the ABC-type 2 sub-family and on a genetic level closely resemble the immunity systems found in lantibiotic producing strains. This group is named after the most prominent member, the CprABC transporter from *C. difficile*, which confers resistance against nisin and gallidermin (McBride and Sonenshein, 2011; Suárez et al., 2013). Here, the NBD is encoded by *cprA* while *cprB* and *cprC* encode the two TMDs. CprB and CprC are of similar size and are predicted to contain six transmembrane helices each. Both CprB and CprC form a functional transporter together in the membrane as a heterodimer (Figure 3).

Other members of this group are *lcrSR-lctFEG* and *nsrFE*_1_*E*_2_*G-XRK*, both present in the genome of *S. mutans* (Figure 2). The encoded TMDs of this group contain six predicted transmembrane helices. They are upregulated via one promoter suggesting that they are expressed in equal stoichiometry. These ABC transporters, are closely related to the immunity ABC transporter LanFEG family, which consists of the NBD LanF and two TMDs LanE and LanG (as an example, the nisin immunity system called NisFEG is highlighted in Figure 2). These LanFEG ABC transporters are co-expressed in lantibiotic producer strains and have been shown to expel lantibiotics from the membrane preventing a suicidal effect (Stein et al., 2003, 2005; Alkhatib et al., 2012; AlKhatib et al., 2014b). The LanFEG genes are, in contrast to the Cpr group, encoded in a larger operon which also include genes for biosynthesis and transport machinery of the produced lantibiotic. Due to the operon similarity, one can assume that the *cpr* operons are evolutionary linked to the producing strains. Here however, only the genes for the resistance proteins are present and none of the biosynthetic machinery. Next to the ABC transporter genes, a TCS is present which consists of the HK and RR, which are distantly located on the chromosome (Suárez et al., 2013) (highlighted in green, Figure 2). These TCSs transfer the stimuli provided by the externally present lantibiotic into the cell and induce transcription of the genes.

## BceAB resistance operon

Lantibiotic resistance operons belonging to the *Bce* group contain genes encoding for an ABC transporter, i.e., two different genes encoding one NBD and one large TMD (highlighted in blue, Figure 2). Additionally, a TCS is present consisting of a histidine kinase and response regulator. BceAB-type (ABC) transporters are putatively involved in antimicrobial peptide as well as lantibiotic removal from the lipid membrane (Gebhard and Mascher, 2011). They have been named after the transporter system from *B. subtilis*, which till date is the best characterized representative of ***B***a***c***itracin ***e***fflux (Bce) transporters, conferring resistance against the antimicrobial peptide bacitracin (Ohki et al., 2003; Rietkötter et al., 2008).

Based on the Transport Classification Database (TCDB), BceAB-type transporters belong to the peptide 7 exporter family (Saier et al., 2009). The BceAB-type transporters are composed of two components, a NBD (BceA) and a single TMD (BceB) (Figure 2). The TMD consists of ten predicted transmembrane helices (TMHs) and contain a large, extracellular domain (ECD_*L*_ where L stands for lantibiotic) between transmembrane helices VII and VIII (Figure 3). This extracellular domain appears to be the hallmark of BceAB-type transporters and consists of ~200–250 amino acids (Ohki et al., 2003; Rietkötter et al., 2008; Khosa et al., 2013; Figure 3).

Bioinformatically, ECD_*L*_ are easy to detect and have been for example identified in the TMDs of the bacitracin resistance-associated ABC transporter BceAB in *Bacillus* species (Rietkötter et al., 2008), in the bacitracin and nisin resistance-associated ABC transporter VraDE in *S. aureus* (Hiron et al., 2011) and the nisin resistance-associated NsrFP in *S. agalactiae* (Khosa et al., 2013). These ECD_*L*_ are found to be crucial for resistance determinants as they are supposed to recognize the lantibiotic extracellularly and subsequently induce the TCS-dependent signal transduction within the cell (Rietkötter et al., 2008; Hiron et al., 2011). Initial substrate binding usually occurs via these ECD_*L*_, however, experimental evidence for this only has been indirectly proven via knockout studies (Falord et al., 2012).

In the Bce group, a BceRS-type TCS has co-evolved (Heijenoort, 1994; Dintner et al., 2011) composed of a response regulator (BceR) and a histidine kinase (BceS). The latter consists of two transmembrane helices with a short extracellular located loop of ~25 amino acids. Such a small loop is unusual for HKs, which normally consists of roughly 115–125 amino acids. This suggested that the TCS lacks an extracellular domain normally present to detect an external stimulus and is therefore, categorized as a member of the intramembrane-sensing histidine kinase family (Mascher et al., 2003; Mascher, 2006). Members of this family have been shown to be responsible for the upregulation of the corresponding ABC-transporter in the presence of its specific lantibiotic (Staron et al., 2011).

In summary, three CprABC-type as well as BceAB-type ABC transporters are mentioned in detail, which are all well studied so far in order to highlight both their functional properties and the differences between these two groups. Within the CprABC group, we have included the NisFEG ABC transporter, which confers immunity against nisin in the producer strains. General characteristics including protein sequence, size, and function of these systems are listed in Tables 1, 2.

**Table 1:** Comparison of the different Cpr-type resistance systems with the TCS and the ABC transporter.

	***CprABCK-R***	***LcrSR-LctFEG***	***NsrFE*_1_*E*_2_*G-XRK***	***NisRK-FEG***
Organism	*C. difficile (Strain630)*	*S. mutans (UA159)*	*S. mutans (UA159)*	*L. lactis (NZ9700)*
Defense category	Resistance	Resistance	Resistance	Immunity
Resistance against	nisin A, subtilin, gallidermin, mutacin 1140, cinnamycin	lacticin 481, nukacin ISK-I	nisin A	nisin A
TMD [aa]	238 (B) 252 (C)	246 (E) 242 (G)	82 (E1) 171 (E2) 248 (G)	242 (E) 214 (G)
Oligomeric state	Dimer (B+C)	Dimer (E+G)	Trimer (G+E1+E2)	Dimer (E+G)
TMHs	6+6	6+6	6+2+4	6+6
NBD [aa]	235	255	234	225
Response regulator [aa]	219	229	219	229
Histidine kinase [aa]	462	437	460	447
Loop-cont. protein	Kinase	Kinase	Kinase	Kinase
Loop size [aa]	113	117	116	112

**Table 2:** Comparison of the different Bce-type resistance machineries with the TCS and the ABC transporter.

	***BceRS-AB***	***BraSR-VraDE***	***NsrFP-RK***
Organism	*B. subtilis (Strain 168)*	*S. aureus (USA300)*	*S. agalactiae (*COH1)
Defense category	Resistance	Resistance	Resistance
Resistance against	bacitracin, actagardine, mersacidin	bacitracin, nisin A, daptomycin	nisin A, nisin H, gallidermin
TMD	646	626	651
Oligomeric state	Monomer	Monomer	Monomer
TMHs	10	10	10
NBD [aa]	253	252	250
Response regulator [aa]	231	221	222
Histidine kinase [aa]	334	295	262
ECD_L_ cont. protein	TMD	TMD	TMD
ECD_L_ size[aa]	216	195	220

## CprABC resistance systems

### The CprABCK-R system from *C. difficile*

The operon of the *cpr* (***c***ationic antimicrobial ***p***eptide ***r***esistance) system from *C. difficile* consists of an ABC transporter and a TCS. The genetic organization of the Cpr system resembles the immunity system found in lantibiotic producing strains (see above).

The CprABC transporter from *C. difficile* is encoded by three different proteins: a nucleotide binding domain called CprA (26 kDa) and the two transmembrane domains called CprB and CprC (27 and 29 kDa, respectively; Figure 3 and Table 1). Both of the TMDs have six predicted transmembrane helices and form a functional heterodimer (Table 1).

The TCS is composed of a RR *cprR* (CD3320) and a HK *cprK* (CD1352). The histidine kinase of the Cpr system contains an extracellular loop (113 aa) (Table 1), which has been proposed to be involved in sensing. Such a loop is a general feature of histidine kinases. The regulator does not directly belong to the *cpr* operon and is distantly located on the chromosome (McBride and Sonenshein, 2011; Suárez et al., 2013).

Within *C. difficile*, the *cpr* system has been shown to confer resistance against several lantibiotics such as nisin A, gallidermin, and subtilin. Although these lantibiotics are quite different in their amino acid composition, however the first two lanthionine rings are structurally conserved in their tertiary structure (Suárez et al., 2013), suggesting the importance of this region for the *cpr* genes to recognize lantibiotics. It was proposed that the lanthionine ring along with the proline and glycine residues form the sequence motif recognized by CprK resulting in signaling of the TCS CprK-CprR (Suárez et al., 2013).

The genes encoding the ABC transporter *cprABC* are regulated by *cprK-cprR* and are found adjacent to *cprK* in the genome. Insertional disruption of one of the transporter genes resulted in significant decrease in resistance against both nisin A and gallidermin. Hence, this TCS and ABC transporter pair contributes to the resistance of *C. difficile* toward many lantibiotics (Suárez et al., 2013). It has been shown that the addition of nisin induced the expression of CprABC, so it could be proven that the CprR is responsible for the upregulation (McBride and Sonenshein, 2011; Suárez et al., 2013).

Additionally, adjacent of the *cprABC* gene cluster, a lipoprotein is present (CD1348). Although, no involvement in lantibiotic resistance has been described so far, the genetic context resembles the BceAB system found in *S. agalactiae*, which contains the *Sa*NSR protein, a membrane associated resistance protein (see below). Interestingly, the lipoprotein is not upregulated by the presence of a lantibiotic or antimicrobial peptide and displays a basal expression level (Suárez et al., 2013).

### The LcrSR-LctFEG and NsrFE_1_E_2_G-XRK resistance systems

*S. mutans* (UA159) has two resistance systems, namely, the LcrSR-LctFEG and NsrFE_1_E_2_G-XRK. Both consist of a TCS and an ABC transporter.

The LcrSR-LctFEG system confers resistance against lacticin 481 and nukacin ISK-I as determined with growth inhibition analyses (Kawada-Matsuo et al., 2013a,b).

Within this system, LcrR (26 kDa) is the RR and the LcrS (50 kDa) is the HK, which also contains an extracellular sensing loop (117aa) (Table 1). The ABC transporter consists of three different domains. The NBD LctF (29 kDa), which is important for the ATP binding and hydrolysis, and two transmembrane domains, LctE (28 kDa) and LctG (27 kDa), which each consist of six transmembrane helices each (Kawada-Matsuo et al., 2013b).

The NsrFE_1_E_2_G-XRK system contains a TCS NsrRK with the NsrR (25 kDa) as RR and the NsrK (53 kDa) as HK containing an extracellular loop of 116 amino acids. The ABC transporter system contains four proteins: the NBD NsrF (26 kDa) and the three TMDs NsrE_1_E_2_G. Here, the NsrG (28 kDa) has six transmembrane helices, the NsrE_1_ (10 kDa) has two and NsrE_2_ (20 kDa) has four transmembrane helices, so in total 12, which is similar to the other known ABC transporters. However, for NsrFE_1_E_2_G resistance against only nisin A was observed, which was examined using deletional mutants within the NsrRK system. For other tested lantibiotics like nukacin ISK-1, no resistance could be observed (Kawada-Matsuo et al., 2013b).

### The NisFEG immunity transporter from *L. lactis*

In the self-immunity system of nisin producing strains, the cytoplasmic NisF (25 kDa) is composed of 225 amino (Siegers and Entian, 1995). Additionally, NisE (28 kDa) and NisG (24 kDa) are predominantly hydrophobic proteins, that form together an integral membrane part of the ABC transporter and are composed of six transmembrane helices each (Siegers and Entian, 1995). Using sequence similarity searches NisFEG likely exhibits a 2:1:1 stoichiometry to form a functional lantibiotic immunity LanFEG transporter (Siegers and Entian, 1995). Various gene knockout studies have shown that out of all the three genes of the ABC transporter, deletion of *nisE* gene has the most detrimental effect on immunity (Siegers and Entian, 1995).

The primarily function of NisFEG in providing immunity to the producer strain is the efflux of nisin molecules from the membrane before they can form pores (Stein et al., 2003; AlKhatib et al., 2014b). A similar function has been identified for the subtilin immunity ABC transporter SpaFEG, which is able to transport subtilin from the cytoplasmic membrane directly back into the exterior (Stein et al., 2005).

When expressed in the nisin sensitive *L. lactis* strain NZ9000, which does not carry the immunity genes *nisI* and *nisFEG* within its genome, NisFEG confers seven to eight fold of immunity when expressed alone (AlKhatib et al., 2014b).

The substrate specificity of NisFEG has been extensively studied. It has been shown that NisFEG recognizes the C-terminally located lanthionine ring and the last six amino acids of nisin as a reduction of 50% in the immunity provided by NisFEG was seen upon deletion of either of them (AlKhatib et al., 2014b).

## The Bce resistance systems

### BceRS-AB system from *B. subtilis*

The BceRS-AB system from *B. subtilis* consists of the ABC transporter, with NBD BceA (28 kDa) and TMD BceB (72 kDa), and the TCS with the response regulator BceR (27 kDa) and the histidine kinase BceS (39 kDa) (Figure 3 and Table 2). Various growth inhibition assays of strains expressing BceRS-AB and several deletion mutants have shown, that this system mediates resistance against actagardine, mersacidin, and bacitracin (Ohki et al., 2003; Staron et al., 2011).

For signal transduction purposes, both the BceAB ABC transporter as well as the TCS need to be present to confer a signal transduction inducing upregulation of the genes encoded on the operon. This is an unusual mode of signal transduction, as the HK cannot sense bacitracin alone, and needs the presence of the ABC transporter to sense the substrate in the surrounding. Furthermore, an ATP hydrolysis deficient transporter mutant highlighted that hydrolysis is required for this signaling process (Rietkötter et al., 2008). Based on random mutagenesis studies it was shown that the C-terminal part of the TMD BceB, specifically up to helix VIII is important for signaling and resistance of the BceAB-RS system in *B. subtilis* (Kallenberg et al., 2013).

The BceAB from *B. subtilis* has been to the best of our knowledge, the only system which has been purified and shown to form a multicomponent complex with its designated TCS BceRS upon binding of bacitracin (Dintner et al., 2014). These biochemical analyses of the BceAB and BceRS proteins showed that the TCS, more specifically the BceS module, and the transporter form a so-called sensory complex in the cytoplasmic membrane, where the kinase activity is relying on the BceAB transporter (Dintner et al., 2014). This further underlines the fact that the BceAB transporter from *B. subtilis* is directly involved in bacitracin sensing and consequently triggers the upregulation of its own gene by the TCS BceRS. This was further highlighted by mathematical modeling response dynamics of the Bce system, which suggested a direct correlation between the transport activity of BceAB, and the BceS kinase signaling activity (Fritz et al., 2015).

BceAB-like transporters are thought to recognize the target-peptide complex within the membrane and not the peptide as such (Bernard et al., 2007; Rietkötter et al., 2008). This idea is further strengthened by experiments suggesting that BceAB of *B. subtilis* does not export bacitracin, but instead acts as a flippase of the target molecule undecaprenyl pyrophosphate (UPP) to the cytoplasmic side of the membrane (Kingston et al., 2014), thereby removing the target of bacitracin. However, Surface Plasmon Resonance (SPR) spectroscopy studies of BceAB have shown that the TMD BceB binds bacitracin with a high affinity (*K*_*D*_ of 60 nM) in detergent solution and appears to be specific for the active bacitracin-Zn^2+^-complexed form (Dintner et al., 2014). Although these results do not rule out that a bacitracin-UPP complex is recognized by BceAB, it suggests an effluxing mechanism for bacitracin. Random mutagenesis studies further highlighted, that the C-terminal part of the TMD BceB up to helix VIII is important for the signaling and the resistance of the BceRS-AB system in *B. subtilis* (Kallenberg et al., 2013).

### The VraDE-BraRS from *S. aureus*

The VraDE-BraRS system of *S. aureus* is a system composed of the NBD VraD (28 kDa), the TMD VraE (70 kDa), the response regulator BraR (25 kDa), and the histidine kinase BraS (34 kDa). This system has been identified in *S. aureus* since only two of the 16 TCSs present have been linked to the Bce family. Here, the TCS GraRS (Meehl et al., 2007) and the VraDE-BraRS system, mediate cationic antimicrobial peptide resistance (Hiron et al., 2011).

The VraDE-BraRS system of *S. aureus* confers resistance against bacitracin, nisin A and daptomycin as determined via growth inhibition experiments of *S. aureus* (Hiron et al., 2011; Popella et al., 2016). Transcriptional fusions using the operon promoter revealed increased expression when induced with increasing sub-lethal bacitracin and nisin concentrations as previously observed for the BceRS/BceAB module of *B. subtilis* (Ohki et al., 2003; Hiron et al., 2011). The TCS BraRS activates transcription of the *BraDE* and *VraDE* operons, encoding two ABC transporters, which play distinct and original roles in bacitracin and nisin resistance. Here, VraDE is a specific detoxification system and is sufficient to confer resistance against bacitracin and nisin when expressed alone (Hiron et al., 2011) whereas BraDE and BraRS are involved in antibiotic sensing and signaling, respectively.

The ABC transporter VraDE is directly involved in bacitracin resistance. By using chimeric ABC transporter and domain-swapping variants, where the extracellular loop of VraD was exchanged by the one of VraE, it was observed that the extracellular domain of VraE is the determinant for bacitracin specificity (Hiron et al., 2011; Popella et al., 2016). Furthermore, it was shown that VraH, a small transmembrane protein, is further an essential component of the VraDE complex to form the functional VraDEH complex. VraH of *S. aureus* JE2 is a positively charged C-terminus containing a conserved YYKRREEKGK motif. The cytoplasmic VraD interacts with the transmembrane protein VraH. This complex however is formed only in the presence of VraE (Popella et al., 2016). Interestingly, VraH is only important for resistance against gallidermin. Nisin and bacitracin resistance appears to be independent of VraH. This is rather unexpected since gallidermin and nisin share the same structural and mechanistic features, whereas bacitracin is structurally unrelated. Additionally, gallidermin does not form pores in the membranes of most bacteria in contrast to nisin, indicating another mode of action (Popella et al., 2016).

The BraRS TCS has been shown to be specific for nisin and no upregulation occurred when using other antibiotics like vancomycin, fosfomycin, oxacillin, colistin, capreomycin, viomycin, or daptomycin (Hiron et al., 2011).

### The NsrFP-RK system from *S. agalactiae*

The NsrFP-RK system from *S. agalactiae* is composed of the NBD NsrF (28 kDa), the TMD NsrP (74 kDa), the RR NsrR (25 kDa), and the HK NsrK (31 kDa) (characteristics of the proteins are listed in Table 2; Khosa et al., 2013). Further, the NsrFP-RK system includes an additional serine protease *Sa*NSR, which inactivates nisin by cleaving off the last six amino acids. *Sa*NSR is anchored in the membrane via a single transmembrane segment (Khosa et al., 2016a). This system confers resistance to multiple lantibiotics such as nisin A, nisin H, and gallidermin as determined with growth inhibition experiments in *L. lactis* (Khosa et al., 2013; Reiners et al., 2017). Furthermore, it was shown by SYTOX-green assay, that the resistance conferred by the ABC transporter NsrFP is imparted by the inhibiting pore formation in the cell membrane and similar to the CprABC system (see above), it also recognizes specifically the N-terminal part of lantibiotics (Reiners et al., 2017), suggesting a common substrate specificity between these systems. For NsrFP, this was shown by a comprehensive mutational analysis of nisin and comparison of the fold of resistance (Reiners et al., 2017), thereby sensitively quantifying and comparing the growth inhibition studies between lantibiotics and their variants (AlKhatib et al., 2014a,b; Reiners et al., 2017). An advantage of the NsrFP system is their heterologous expression in *L. lactis*, which allows a mutational analysis, and holds true for the predominant substrate nisin A. Furthermore, the corresponding NsrR (RR) and NsrK (HK) are not present, which allowed the characterization of the NsrFP ABC transporter alone.

For NsrFP, an efflux transport activity has been reported (Reiners et al., 2017). Here, a peptide release assay revealed the transport direction of NsrFP. The efflux of nisin was shown by the amount of nisin present in the supernatant of the cell culture in comparison to a strain harboring an empty plasmid as well as a transport deficient mutant of NsrFP. Furthermore, NsrFP is the BceAB-type transporter that actually suggests an efflux transport direction using a peptide release assay. These results are similar to those previously obtained for the lantibiotic immunity transporters NisFEG and SpaFEG from *L. lactis* and *B. subtilis*, respectively, which have been shown to export their corresponding lantibiotics (Stein et al., 2003, 2005).

On the contrary, the TCS NsrRK of the Nsr system has been poorly described so far. Here, NsrR belongs to the large OmpR/PhoB subfamily of response regulators. The structure of the regulator NsrR has been solved by X-ray crystallography and a model in active dimeric DNA-bound state was postulated (Khosa et al., 2016b). This model revealed that the amino acids involved in phosphorylation, dimerization, as well as DNA-binding are conserved on sequence level throughout the family of regulators found in the BceAB resistance systems identified, so far. This suggests that other BceAB-type response regulators will probably have a similar tertiary structural arrangement.

An extra feature of the NsrFP-RK system is the presence of a membrane-associated serine protease in the operon called *Sa*NSR, which cleaves nisin at its C-terminus and the product nisin_1−28_ has been shown to be 20–100 fold less effective against Gram-positive bacteria membranes (Sun et al., 2009; Khosa et al., 2016a).

The structure of *Sa*NSR was solved at 2.2 Å resolution and displays an N-terminal helical bundle, a protease cap and core domain. Within the latter, the highly conserved TASSAEM region is present. This region contains the active site and lies in a hydrophobic tunnel. Extensive computational modeling of the *Sa*NSR/nisin complex revealed that *Sa*NSR specifically recognizes the C-terminus of nisin, more specifically the last two lanthionine rings of nisin ensuring the exact coordination of the nisin cleavage site at the TASSAEM region (Khosa et al., 2016a). This clearly indicates that in contrast to the efflux mechanism of the ABC transporter NsrFP, *Sa*NSR is highly specific to confer resistance solely against nisin A.

## Future directions

Since (multi-) antibiotic resistant bacteria have rapidly evolved during the last decades, the urgent need for novel compounds is increasing. The secretion of antimicrobial peptides by microorganisms represent a giant pool of novel compounds, which can be used as initial lead structures to develop novel antibiotics.

Here, lantibiotics as small ribosomally-synthesized antimicrobial peptides became relevant and due to genome sequencing the number of identified lantibiotics is rapidly growing. Lantibiotics bind to the essential pyrophosphate-sugar moiety of the cell wall precursor lipid II. This is in contrast to well-known glycopeptide antibiotics vancomycin and teicoplanin, which bind to the D-Ala-D-alanyl group of lipid II (Draper et al., 2015). Due to this, it is believed that new resistance mechanisms against lantibiotics are hard to establish for bacteria.

However, inherent resistance against lantibiotics and antimicrobial peptides are already present and are mediated by ABC transporters, Cpr- and Bce-type transporter, which are present in most human pathogenic bacterial strains. This hampers a wide usage of lantibiotics against severe bacterial infections.

The Cpr ABC transporters resembles the known LanFEG transporter found in lantibiotic producer strains, involved in (auto-)immunity suggesting an evolutionary link. In contrast, the Bce-type ABC transporters appear to be a novel and unique transporter family, interacting directly with the TCS in the presence of the lantibiotic (Khosa et al., 2013; Dintner et al., 2014). Mechanistically, both families are not very well-understood. For the Cpr systems, it has been observed that they are able to expel the lantibiotic from the membrane back into the extracellular media. This would suggest that inhibiting the transporter would allow the lantibiotic to penetrate the membrane again. Therefore, a compound specifically targeting the Cpr transporters would be ideal to use as a lead compound ensuring the potent activity of the lantibiotic itself. To achieve this, more knowledge has to be gained about the exact mechanism of these transporters. Although studies have been performed *in vivo*, the understanding of binding affinities of the lantibiotic toward the transporter as well as some structural studies will clearly be needed in future. Structurally, the Cpr transporter appears to be a different class of ABC transporter since none of known ABC transporter structures seems to be an useful template for molecular modeling approaches using available computer tools, which suggest that structural studies will be needed.

The mechanism of the Bce-type transporters is poorly understood. Recently, for the NsrFP system from *S. agalactiae*, an export function was reported using a peptide release assay (Reiners et al., 2017). Nevertheless, also a flippase activity of the target molecule lipid II of the ABC transporters would fit to the published studies, since the amount of the lantibiotic in the supernatant would also increase, if the target of the lantibiotic is not present anymore. This hypothesis is also in line with the studies of the BceAB transporter of *B. subtilis* by Kingston et al. (2014). This flippase activity would also explain why these Bce-type transporters appear to have a large substrate spectrum and are able to confer resistance against structurally different lantibiotics as well as some antimicrobial peptides. Here a special focus might be present for the uncharacterized ECD_*L*_, the hallmark of BceAB transporters. The function of this ECD_*L*_ is unknown, the structure remains elusive so far and further it is not proven that it interacts with the lantibiotic. Therefore, studies revealing function of this ECD_*L*_ would likely give a starting point for studies toward the identification of an inhibitor. Remarkable is the complex formation of the BceAB transporter with the TCS. This unusual partnership within the membrane results in a macromolecular complex, which is induced by the presence of the lantibiotic. A pioneering study of the BceAB system from B. subtilis suggests that a bacitracin-UPP complex is recognized by BceAB, recognized by the C-terminal part of the TMD BceB up to helix VIII (Kallenberg et al., 2013). Also the complex with the TCS has been shown to be at least stabilized via the TMD. Therefore, studies on the exact function of the ECD_*L*_, both biochemically and structurally, will be needed to gain a full understanding of the BceAB system.

Both transporters (BceAB and CprABC) have in common that they are upregulated by a specific TCS induced by the peptide in the medium. Inhibiting the histidine kinase would therefore, be an excellent target for novel drugs, which then in combination with lantibiotics would be a treatment procedure.

Since lantibiotics are active in the low nanomolar range against strains without resistance mechanisms, their potential is clearly demonstrated. If the resistance mechanisms of both transporter families are understood, the first step to inhibit these has been taken, lantibiotics and its variants thereof will be able to fulfill their whole antimicrobial potential.

With novel lantibiotics being identified in new sequenced genomes, the full potential of these antimicrobial peptides has likely not been explored. Probably, for every new lantibiotic discovered a specific resistance system may be present in some human pathogens. Since all lantibiotics target a similar molecule within the target membrane, it is plausible that these resistance mechanisms will be similar to the Cpr and Bce systems, therefore they serve as model systems for lantibiotic resistance.

## Author contributions

RC, JZ-K, and SS wrote the manuscript. RC and SK prepared the figures. RC, JZ-K, SK, and SS finalized the manuscript.

### Conflict of interest statement

The authors declare that the research was conducted in the absence of any commercial or financial relationships that could be construed as a potential conflict of interest. The reviewer CC and handling Editor declared their shared affiliation.
